# Invisible scars, collapsed trust: a mixed-methods inquiry into the cognitive-empathic divide in school bullying and a systemic intervention framework

**DOI:** 10.3389/fpsyg.2026.1765331

**Published:** 2026-04-22

**Authors:** Binbin Mao, Jinan Jia

**Affiliations:** 1 College of educational Science, Inner Mongolia Minzu University, Inner Mongolia, Tongliao, China; 2 Inner Mongolia Student Bullying Prevention Research center, Inner Mongolia, Tongliao, China; 3 College of Foreign Languages, Inner Mongolia Minzu University, Inner Mongola, Tongliao, China

**Keywords:** cognitive-empathic divide, moral disengagement, quantitative and qualitative research, restorative justice, school bullying, social emotional learning

## Abstract

**Introduction:**

School bullying remains a persistent global challenge despite extensive policy implementation. This study investigates the Cognitive-Empathic Divide defined as the discrepancy between students’ subjective experiences of trauma and the objective, behavior-based frameworks utilized by institutional authorities.

**Methods:**

Utilizing an explanatory sequential mixed methods design, including 7,341survey respondents and 25 semi-structured interviews.

**Results:**

Quantitative results confirmed a Coping Paradox (p <.001) among high-risk groups, indicating high frequency but maladaptive help seeking behaviors. Qualitative inquiry, conducted via reflexive thematic analysis, elucidated a cyclical process of Seek Help-Disappointment-Abandonment driven by institutional invalidation. Furthermore, an empirical analysis of 155 civil judgments provided evidence of institutional risk avoidance cultures that marginalize nonphysical harm.

**Discussion:**

The findings advocate for a paradigm shift from behavior centric management to trauma informed institutional cultures. A systemic intervention framework is proposed, integrating Social Emotional Learning and Restorative Justice within a Whole School Approach to bridge the cognitive empathic divide and rebuild institutional legitimacy.

## 1 Introduction

School bullying is recognized as a significant international challenge, with global statistics indicating that every month, one in three learners experiences some form of bullying (UNESCO, 2024). Despite a proliferation of policy documents and intervention programs, the prevalence and associated psychological distress have not been consistently mitigated (Luo et al., 2022). This research posits that the current impasse stems from an epistemological rupture a discrepancy between students’ phenomenological reality of emotional pain and the positivistic, evidence-based frameworks adopted by school authorities (Levitt et al., 2018).

The Cognitive-Empathic Divide reflects a disconnect between the two primary dimensions of empathy: cognitive empathy (the ability to recognize mental states) and affective empathy (the drive to appropriately respond to those states) (van Noorden et al., 2015; Arslan et al., 2021). Students typically experience bullying through a feeling of insult, but an institutional response remains strictly at the cognitive level (e.g., asking Is there proof?), it effectively fails to enter the victim’s domain of affective empathy, leading to secondary victimization—defined here as the additional psychological harm caused by invalidating institutional responses.

Recent meta-analytical evidence confirms a critical bidirectional relationship: low empathy predicts future bullying, and the act of bullying itself predicts subsequent declines in empathy (Chu, 2025). Consequently, an institutional framework that ignores the affective dimension of trauma may institutionally demonstrate and legitimize the emotion-stripped cognitive framework that drives bullying behavior. Bridging this divide is a prerequisite for effective intervention, requiring a shift from behavior-based definitions to trauma-informed perspectives rooted in subjective psychological trauma—the internal reality of emotional distress and traumatic psychological fixation created by victimization (Byrne, 2021; Maier and Seligman, 1976).

In the process of analyzing the factors associated with school bullying, we identified a cognitive-empathic divide between students’ subjective perceptions of trauma and adults’ objective behavior-based assessment frameworks. Building on this finding, we further examined the characteristics and formation mechanisms of this cognitive-empathic divide, and attempted to develop a culturally adaptive intervention framework, which is expected to exert a positive impact on the prevention and management of school bullying.

## 2 Materials and methods

### 2.1 Phase 1: quantitative validation

A cross-sectional survey was conducted with 7,341 students from eight cities and leagues in the Inner Mongolia Autonomous Region, China, namely Hohhot, Ulanqab, Hulunbuir, Tongliao, Chifeng, Xilingol League, Ordos, and Bayannur. In each city, we selected one junior high school, one senior high school, and one primary school as the research sites for our study. Students from Grade 4 in primary school to Grade 2 in senior high school as research participants., from June 8 to10, 2025.

#### 2.1.1 Grouping criteria

High-risk groups were defined using the top 30th percentile of scores from the Bullying Victimization Questionnaire and the Aggression Questionnaire. The High-Victimization group consisted of 2,186(Low: N = 5,155).

#### 2.1.2 Missing data handling

The High-Aggression group comprised 2,202 and the Low-Aggression group 4,320 (Total N = 6,522). The 819-case discrepancy is attributed to structural missing data, specifically incomplete or invalid responses within the Aggression Scale sub-dimensions, which were excluded from that specific sub-analysis to maintain integrity.

#### 2.1.3 Measures

Operationalized using the Chinese Student Emotional Competence Questionnaire (EQ), Moral Disengagement Scale, and Coping Style Questionnaire.

The Chinese Student Social and Emotional Competence Questionnaire (Middle School Version), developed by Beijing Normal University and the Ministry of Education-UNICEF Social Emotional Learning (SEL) Project Team. The scale comprises six dimensions: self-awareness, self-management, other-awareness, other-management, collective-awareness, and collective-management. It demonstrated excellent internal consistency with a Cronbach’s alpha coefficient of 0.95 for the total scale and 0.84–0.91 for each subscale (CASEL, 2020; Durlak et al., 2011). The Chinese version of the Moral Disengagement Scale was revised by Wang and Yang (2009) based on the original questionnaire developed by Bandura et al. (1996). The scale comprises eight mechanisms of moral disengagement, each containing four items, yielding a total of 32 items. Eight mechanisms:moral justification, euphemistic labeling, advantageous comparison, displacement of responsibility, diffusion of responsibility, distorting consequences, dehumanization, attribution of blame. The Cronbach’s alpha coefficient of the total scale was 0.87, and the split-half reliability coefficient was 0.80. The coefficients of each dimension were between 0.68 and 0.82, indicating that the questionnaire had good reliability and structural validity. The Coping Style Questionnaire, Developed by Guo et al. (2016), which consists of 5 dimensions: Problem-solving, Help-seeking Fantasy, Self-blame and Rationalization, The test–retest reliability coefficient of the scale was 0.89 (Latimer et al., 2005; Mahoney et al., 2018).

### 2.2 Phase 2: qualitative descriptive inquiry

The study utilized a qualitative descriptive design centered on Reflexive Thematic Analysis (RTA) (Braun and Clarke, 2021).

#### 2.2.1 Participants

Following a large-scale questionnaire survey. We conducted targeted sampling to recruit participants who scored higher on the bullying victimization subscale for semi-structured interviews, including 15 students:self-identified victims, bystanders, or perpetrators and 10 teachers, from June 11to12, 2025. The teacher participants in the interviews were mainly recruited from three groups: school administrators, head teachers, and mental health teachers.

#### 2.2.2 Sample size justification

To justify the sample size of 25, this study eschews the positivistic concept of data saturation (which is incongruent with a reflexive, non-positivistic paradigm (Braun and Clarke, 2021). Instead, it adopts the information power standard (Malterud et al., 2016), which is more aligned with the study’s paradigm. This standard posits that the more information-rich the sample holds, the fewer participants are needed. Given the study’s clear aim, highly specific purposive sampling, and the rich, deep, and reflective experiences of the participants, the 25 sample was deemed to have high information power and was fully sufficient to elucidate the complex phenomenological questions under investigation.

#### 2.2.3 Information power

Sample size was justified using the five dimensions of information power (Malterud et al., 2016): (1) *Aim:* Narrowly focused on the cognitive-empathic divide; (2) *Specificity:* Participants had direct bullying experience; (3) *Theory:* Grounded in SEL and RJ frameworks; (4) *Dialogue:* In-depth, semi-structured interviews yielded reflective narratives; (5) *Strategy:* Cross-case interpretive analysis.

#### 2.2.4 Trustworthiness

Rigor was maintained through researcher reflexivity and an audit trail. Participant feedback was utilized as a tool for collaborative reflection rather than a positivist validity check (Lincoln and Guba, 1985).

## 3 Results: the collapsed circle of trust

### 3.1 The coping paradox and learned helplessness

A key finding was the Coping Paradox. Both High-Victimization (*p* = 0.013) and High-Aggression (*p* < 0.001) groups scored significantly higher on the Coping Style Total Score (including Help-Seeking) than control groups. However, this increased coping effort was significantly negatively correlated with Student Emotional Competence (r = −0.258, p < 0.001).

Their high Help-Seeking scores capture the initial phase of the cycle, but these attempts are met with systemic invalidation, through interview data, it is shown that the students’ views are, telling the teacher will only make things worse, leading to the depletion of psychological resources. This provides powerful verification of learned helplessness within the school system (Maier and Seligman, 1976).

### 3.2 Moral disengagement and the aggressor

The High-Aggression group scored significantly higher on Moral Disengagement (d = −0.383, *p* < 0.001). Correlation analysis showed a significant negative correlation between Moral Disengagement and Emotional Competence (r = −0.331), suggesting that moral disengagement serves as a cognitive mechanism linking low emotional competence with aggressive behavior.

### 3.3 The phenomenology of harm (qualitative findings)

The Student Definition: Subjective Feeling. Interview data consistently show that for students, the non-negotiable standard for defining bullying is the emotional impact. One student articulated, If a person’s joke already makes you feel like a burden, it is already bullying. This feeling-based definition expresses a legitimate pain that demands validation (Copeland et al., 2013).

### 3.4 The institutional definition: objective evidence

In contrast, teachers tended to prioritize objective evidence. Interviews revealed that adults frequently minimize relational aggression (e.g., exclusion) because it is invisible (Hong and Espelage, 2012). This aligns with recent findings by López-Castedo et al. (2024), who noted that teachers often view mediation as the primary intervention. However, applying mediation to a power imbalance can constitute systemic invalidation, falsely implying equal responsibility (Thompson and Smith, 2011).

### 3.5 Integration: joint display of the epistemological rupture

Following APA JARS-Mixed standards, the integration of perspectives is presented in Table 1.

**Table 1:** Joint display of bullying definitions and meta-inferences.

Behavior type	Student theme (subjective)	Teacher theme (objective)	Meta-inference (integration)
Verbal insults	Core is the feeling of insult and mental harm.	Often dismissed as joking or general conflict.	Institutional systems underestimate verbal harm due to an evidence-bias (22.1% reported verbal bullying).
Social exclusion	Severe due to power imbalance and loneliness.	Difficult to define; misunderstood as unsociable.	Relational aggression is systematically under-detected due to its covert nature.
Physical conflict	Bullying defined by the emotional block.	Identified only if physical injury is visible.	Over-reliance on physical markers devalues the victim’s affective reality.

This table synthesizes qualitative findings to visually illustrate the cognitive discrepancies between students (subjective experiences) and teachers (objective assessments) regarding three core bullying behaviors and derives integrated meta-inferences to reveal systemic blind spots in the identification of school bullying.

## 4 Discussion

### 4.1 Mechanisms of aggression and seek help–disappointment–abandonment cycle

Quantitative data confirm that the High-Aggression group scored significantly higher on Moral Disengagement than the low-risk group (M = 63.90 vs. M = 56.52, *p* < 0.001, d = −0.383). Qualitative narratives identified a Victim-Perpetrator power compensation cycle, where victims adopt moral disengagement as a maladaptive response to regain control by suppressing guilt (Zhang and Wu, 2025). The qualitative data establishes a vicious cycle modeled as follows: Seek help, the student reports the behavior. Disappointment, the adult responds with invalidation (It’s just a joke) or blame, causing secondary victimization. Abandonment, the student learns that help-seeking is futile. This silence is driven by a profound sense of futility rather than simple fear. Recent research suggests this abandonment phase is linked to moral disengagement, where victims may give up on self-protection and abandon the self (Zhang and Wu, 2025). School bullying is not merely a behavioral issue but a systemic failure of empathy. The identified Coping Paradox validates the theory that high-risk students possess the motivation to seek help but lack the institutional trust to receive effective validation (Byrne, 2021). This aligns with recent research suggesting that Whole School Approach (WSA) interventions, while reducing observable behaviors like cyberbullying (by 25%), often fail to improve internal states like anxiety (Roshini et al., 2025; Fulhman et al., 2023).

### 4.2 Judicial and managerial evidence

Analysis of 155 civil judgments in China provided empirical support for institutional paralysis. Schools were held liable in only 32.9% of cases (Gao et al., 2025). There was a significant positive correlation (*p* < 0.05) between school inaction and the severity of psychological harm, suggesting that school passivity exacerbates trauma. This reflects a Judicial Cognitive-Empathic Divide, where psychological harm (affecting 74.4% of cases) is often viewed as no big deal by judges compared to physical injury (Zhao and Zhang, 2025; Copeland et al., 2013; Wolke and Lereya, 2015).

### 4.3 Systemic intervention framework: SEL-RJ-WSA

#### 4.3.1 Pillar 1: social emotional learning (SEL)

SEL should be targeted rather than universal. Evidence from China suggests it is most effective for high-risk subgroups like left-behind children (Li et al., 2024; CASEL, 2020; Durlak et al., 2011). SEL can be used to build empathy and counteract moral disengagement for the high-level bullies and enhance psychological resilience, develop social competencies, and foster the recognition of their inherent character strengths for the bullying victims (Balasooriya Lekamge et al., 2025).

#### 4.3.2 Pillar 2: restorative justice (RJ)

RJ resonates with traditional Chinese values of Harmony (*hé wéi guì*). By fostering a sense of shame and self-correction, RJ achieves deeper behavioral change than punitive measures (Lui, 2024; Fulhman et al., 2023).

#### 4.3.3 Pillar 3: whole school approach (WSA)

WSA must move beyond performative prevention, ensure that reporting mechanisms do not stop at mediation but trigger appropriate SEL support and RJ processes. This structural shift is essential to protect teachers and students from the pressure of reputation concerns (Slee, 1995; Solarz, 2008; Song et al., 2024).

## 5 Limitations

This study is subject to several constraints. First, the cross-sectional quantitative design precludes causal inferences regarding institutional influence on behavior. Second, the qualitative sample was restricted to two schools in a single city in Inner Mongolia, limiting the transferability of findings to all Chinese regional contexts. Third, the reliance on self-report measures may introduce social desirability bias. Finally, the 819-case discrepancy in the aggression data suggests potential participant fatigue that future longitudinal studies should address using multi-source data.

## 6 Conclusion

Bridging the cognitive-empathic divide requires a paradigm shift from behavior management to cultural transformation. By acknowledging invisible scars as legitimate harm and implementing the integrated SEL-RJ-WSA framework, schools can rebuild institutional trust and ensure a psychologically safe environment for all students.

## Data Availability

The original contributions presented in the study are included in the article/supplementary material, further inquiries can be directed to the corresponding author.
